# N-cadherin mediates the migration of bone marrow-derived mesenchymal stem cells toward breast tumor cells

**DOI:** 10.7150/thno.59703

**Published:** 2021-05-03

**Authors:** Sanghyuk Choi, Jinyeong Yu, Wootak Kim, Ki-Sook Park

**Affiliations:** 1 Graduate School of Biotechnology, Kyung Hee University, Yongin 17104, Republic of Korea.; 2 Department of Biomedical Science and Technology, Graduate School, Kyung Hee University, Seoul 02447, Republic of Korea.; 3 East-West Medical Research Institute, Kyung Hee University, Seoul 02447, Republic of Korea.

**Keywords:** bone marrow-derived mesenchymal stem cell, breast tumor, tumor microenvironment, N-cadherin, TGF-β.

## Abstract

**Rationale:** Bone marrow-derived mesenchymal stem cells (BM-MSCs) recruited into breast tumors regulate the behavior of tumor cells via various mechanisms and affect clinical outcomes. Although signaling molecules, such as transforming growth factor β (TGF-β), are known to transmit signals between BM-MSCs and breast tumor cells for recruiting BM-MSCs, it is unclear which specific intrinsic molecules involved in cell motility mediate the migration of BM-MSCs into breast tumor. It is also unclear as to how specific intrinsic molecules contribute to the migration.

**Methods:** Conditioned medium (CM) from breast tumor cells (MCF-7 and MDA-MB-231) that simulates breast tumor secreting TGF-β was used to examine the migration of BM-MSCs into breast tumors. A three-dimensional migration assay was performed to investigate the collective migration of BM-MSCs, maintaining cell-cell adhesion, toward breast tumor cells.

**Results:** N-cadherin formed adherens junction-like structures on the intercellular borders of BM-MSCs, and TGF-β increased the expression of N-cadherin on these borders. Knockdown of Smad4 impaired the TGF-β-mediated increase in N-cadherin expression in BM-MSCs, but inhibitors of non-canonical TGF-β pathways, such as extracellular signal‐regulated kinases, Akt, and p38, did not affect it. siRNA-mediated knockdown of N-cadherin and Smad4 impaired the migration of BM-MSCs in response to TGF-β. Conditioned medium from breast tumor cells also enhanced the expression of N-cadherin in BM-MSCs, but inactivation of TGF-β type 1 receptor (TGFBR1) with SB505124 and TGFBR1 knockdown abolished the increase in N-cadherin expression. BM-MSCs collectively migrated toward CM from MDA-MB-231 *in vitro* while maintaining cell-cell adhesion through N-cadherin. Knockdown of N-cadherin abolished the migration of BM-MSCs toward the CM from breast tumor cells.

**Conclusion:** In the present study, we identified N-cadherin, an intrinsic transmembrane molecule in adherens junction-like structures, on BM-MSCs as a mediator for the migration of these cells toward breast tumor. The expression of N-cadherin increases on the intercellular borders of BM-MSCs through the TGF-β canonical signaling and they collectively migrate in response to breast tumor cells expressing TGF-β via N-cadherin-dependent cell-cell adhesion. We, herein, introduce a novel promising strategy for controlling and re-engineering the breast tumor microenvironment.

## Introduction

Breast tumor cells dynamically interact with the tumor microenvironment consisting of various non-tumor cells, including cancer-associated fibroblasts (CAFs), endothelial cells, adipocytes, and immune inflammatory cells 1-4. CAFs are heterogeneous in their origin; they may originate from quiescent fibroblasts residing in the host-resident tissue, endothelial cells, pericytes, epithelial cells, and mesenchymal stem cells recruited from a distant source, such as bone marrow 5-7. Similar to cells in wounds that recruit bone marrow-derived mesenchymal stem cells (BM-MSCs) for healing wounds, cells in different types of tumors, including those of ovary, pancreas, prostate, gastrointestinal tissue, and breast, recruit mesenchymal stem cells and progenitors from the bone marrow, and the BM-MSCs recruited into tumors differentiate into CAFs that promote metastasis and proliferation of tumor cells 2, 4, 8-17. Various molecules, interleukin 17B (IL-17B) and transforming growth factor β (TGF-β), are involved in the recruitment of BM-MSCs into tumor microenvironment, and most of these molecules are also involved in the activation of CAFs following the differentiation of BM-MSCs into CAFs 2, 14, 17-19.

The human TGF-β family consists of 33 members that are classified into several subfamilies, including TGF-βs (TGF-β1, -β2, and -β3), bone morphogenetic proteins (BMPs), growth differentiation factors (GDFs), and activins 20. The TGF-β subfamily ligands are recognized by heterodimeric receptor complexes composed of two classes of serine/threonine kinase receptors—the type 1 and type 2 receptors—and bring the receptors together 20. The constitutively active type 2 receptor then phosphorylates the type 1 receptor and the activated type 1 receptor phosphorylates Smad2/3, which subsequently forms a complex with Smad4 20. This Smad complex regulates the transcription of TGF‐β target genes following entry into the nucleus to transduce the canonical TGF‐β signal 20, 21. TGF‐β signal can be non-canonically transduced independently on Smad proteins through various signaling pathways, such as extracellular signal-regulated kinases (ERKs), Akt, and p38 21-23.

TGF-β ligands play important roles in the regulation of tumor initiation, progression, and metastasis 24 and the plasma level of TGF-β1 ligand increases in patients with breast cancer and decreases after surgical removal of the tumor 25. Different responses of breast tumor cells to TGF-β have been reported 26. TGF-β signaling in tumor cells enhances tumorigenesis by stimulating the epithelial-mesenchymal transition (EMT), invasion, and metastasis of breast tumor cells 27-29. Moreover, the activation of TGF-β signaling is associated with the induction of the cancer stem cell phenotype in breast tumor cells 30-33. However, the loss of TGF-β signaling in tumor cells is associated with an increase in both lung metastasis and infiltration of bone marrow-derived myeloid cells that enhance tumor cell invasion and metastasis 34, 35. TGF-β signaling has also been shown to be intrinsically important in non-tumor cells in the breast tumor microenvironment 26, 27. This TGF-β-mediated regulation of tumor microenvironment is critical for the identification of TGF-β as a tumor suppressor or promoter 26, 27. For example, TGF-β activates CAFs to stimulate the synthesis and secretion of growth factors, cytokines, and ECM molecules that collectively form a tumor-promoting microenvironment 27. However, it is still unclear whether breast tumor cell-derived TGF-β ligands modulate the recruitment of BM-MSCs into breast tumor.

BM-MSCs are multipotent adult stem cells 36, 37 that contribute to the maintenance of bone homeostasis 38 and formation of a niche for hematopoietic stem cells inside the bone marrow 39, 40. BM-MSCs also participate in the repair and regeneration of injured peripheral tissues 41. BM-MSCs are partially derived from the neural crest. Cells from the neural crest that forms during the early embryonic development in vertebrates are very motile to migrate to their final destination where they differentiate into a variety of cells 42, 43. Similar to the neural crest cells, BM-MSCs are migratory. They can migrate into injured tissues to participate in regeneration 41, 44. BM-MSCs migrate in response to various molecules, including stromal cell‐derived factor‐1 (SDF‐1) and TGF-β, secreted by injured tissues 45-47. In our previous studies, we demonstrated that BM-MSCs migrate in response to TGF-β1 in a manner dependent on neural cadherin (N-cadherin), a cell-cell adhesion molecule 48, 49.

Cadherins are a large superfamily of transmembrane proteins, which include N-cadherin, epithelial cadherin (E-cadherin), osteoblast cadherin (OB-cadherin), and vascular endothelial cadherin (VE-cadherin) 50. They mediate intercellular adhesion to neighboring cells via calcium-dependent hemophilic and cadherin type-specific interactions in the extracellular domain 50. β-Catenin and p120 interact with the cytoplasmic domain of cadherins and are physically and functionally connected with the actin cytoskeletal system through ɑ-catenin to form adherens junctions 50. Cadherin-mediated cell-cell adhesion controls important biological processes, such as cell sorting, cell rearrangement, and cell movement, as well as intercellular interactions between neighboring cells 50. E-cadherin and N-cadherin play important roles in the collective directional migration of different cell types 51-53. It remains to be determined whether N-cadherin present on BM-MSCs mediates the collective migration of BM-MSCs toward the tumor microenvironment in a cell-cell adhesion-dependent manner, although we previously demonstrated that N-cadherin mediates the migration of BM-MSCs to TGF-β. In the present study, we examined the migration of BM-MSCs toward breast tumor cells expressing TGF-β. TGF-β increased the expression of N-cadherin on the borders between BM-MSCs in a Smad4-dependent manner. N-cadherin-dependent cell-cell adhesion is required for the migration of BM-MSCs in response to conditions mimicking those prevalent in breast tumor.

## Methods

### Cell lines and cell culture

Human BM-MSCs were obtained from Lonza (Basel, Switzerland) and cultured in Mesenchymal Stem Cell Growth Medium (Lonza). TrypLE (Invitrogen, Carlsbad, CA, USA) was used for subculture, and the medium was changed every 3 days. For all experiments, BM‐MSCs between passages 5 and 7 were used. Dulbecco's Modified Eagle's medium (DMEM; GE Healthcare Life Sciences, Logan, UT, USA) supplemented with heat-inactivated fetal bovine serum (FBS; Invitrogen), 1% L-glutamine (Invitrogen), and 1% penicillin and streptomycin (P/S; Invitrogen) was used for migration experiments using BM‐MSCs. MDA-MB-231 cells were obtained from the American Type Culture Collection (ATCC; Manassas, VA, USA). MDA-MB-231 cells were cultured in DMEM/high glucose (DMEM/high, GE Healthcare Life Sciences), supplemented with 10% FBS and 1% P/S. MCF7 cells were obtained from the Korean Cell Line Bank (Seoul, South Korea). MCF7 cells were cultured in DMEM:Nutrient Mixture F-12 (DMEM/F-12; 1:1; Invitrogen), supplemented with 10% FBS and 1% P/S. All the cells were maintained at 37 °C in a humidified incubator containing 5% CO_2_.

### Reagents

Recombinant human TGF-β1 (referred to as TGF-β in this study) was purchased from R&D Systems (Minneapolis, MN, USA). Ethylene glycol-bis(2-aminoethylether)-N,N,N′,N′-tetraacetic acid (EGTA) was obtained from Sigma (St. Louis, MO, USA). Calcein AM was obtained from Cayman Chemical (Ann Arbor, MI, USA). The following cell signaling inhibitors were used: SB203580 (10 μM; EMD Millipore, Burlington, MA, USA), SB431542 (10 μM; Tocris, Bristol, UK), SB505124 (500 nM, Sigma), LY294002 (2 μM, EMD Millipore), and PD98059 (10 μM, EMD Millipore). All the reagents were reconstituted according to the manufacturer's instructions.

### Preparation of bone marrow cell suspension and colony-forming unit fibroblast (CFU-F) assay

All animal experiments were approved by the Animal Experiment Ethics Committee of Kyung Hee Hospital Medical Center (KHMC-IACUC-2019-014) and performed according to the Institutional Animal Care and Use Committee (IACUC) guidelines. Eight-week-old male C57BL/6 mice (DBL, Seoul, South Korea) were used in all the animal experiments. Femurs and tibias were resected and crushed with a pestle. The crushed bones were washed twice using a Hanks' balanced salt solution supplemented with 2% FBS (Invitrogen) and 1% P/S (Invitrogen). The bone fragments were collected and incubated for 1 h at 37 °C in DMEM (Invitrogen) containing 0.2% collagenase (Wako Chemicals USA, Richmond, VA, USA) and 1% P/S (Invitrogen) with occasional agitation. The suspension was filtered through a 40-μm cell strainer (BD Biosciences, San Jose, CA, USA) to remove debris. The cell suspension was centrifuged at 300 × *g* for 10 min at 4 °C. The cell pellet, thus obtained, was washed with Minimum Essential Medium Eagle Alpha Modification (α-MEM; Sigma) containing 20% FBS (Invitrogen), 1% L-glutamine (Invitrogen), 5% pyruvate (Invitrogen), and 1% P/S (Invitrogen). The cells were then seeded in 24-well plates at a density of 1.5 × 10^4^ cells/well using the medium used for washing the cell pellet. After 10 days, cell colonies were fixed with 4% paraformaldehyde (PFA; 3M Science, Saint Paul, MN, USA) in phosphate-buffered saline (PBS) for 10 min on ice after washing three times with ice-cold PBS. Immunocytochemistry for N-cadherin was performed using a primary antibody for N-cadherin (1:30, Invitrogen) according to standard protocols.

### Conditioned medium

MDA-MB-231 and MCF7 cells were cultured in DMEM/high supplemented with 10% FBS and 1% P/S and DMEM/F-12 (1:1) supplemented with 10% FBS and 1% P/S, respectively. After culturing to confluence, the cells were rinsed with PBS and incubated with DMEM/high supplemented with 1% P/S for 3 days. To prepare conditioned medium (CM) for the control, DMEM/high supplemented with 1% P/S was incubated for 3 days under cell-free conditions. Conditioned media from MDA-MB-231 (MDA CM), MCF7 (MCF7 CM), or the control (CON CM) were filtered through a 0.2-μm filter (Corning, NY, USA), aliquoted, and stored at -80 °C before use.

### Transfection of cells with small interfering RNAs (siRNAs)

BM-MSCs were seeded in 6-well plates at a density of 1.3 × 10^5^ cells/well and incubated in the culture medium for 24 h. BM-MSCs were then transfected with the siRNAs using Lipofectamine RNAiMax reagent (Invitrogen) under conditions of serum starvation. The siRNAs used were as follows: scrambled negative control siRNAs (Origene, Rockville, MD, USA), N-cadherin siRNAs (Invitrogen), Smad4 siRNAs (sense: 5′-GAGUAAUGCUCCAUCAAGUUU-3′; antisense: 5′-ACUUGAUGGAGCAUUACUCUU-3′; Genolution, Seoul, South Korea), TGF-β type 1 receptor (TGFBR1) siRNAs #1 (sense: 5′-GAGAAAGUGGCCAUUUACAUU-3′; antisense: 5′-UGUAAAUGGCCACUUUCUCUU-3′; Genolution), TGFBR1 siRNAs #2 (sense: 5′-GGGUCUGUGACUACAACAUUU-3′; antisense: 5′-UGUUGUAGUCACAGACCCUU-3′; Genolution), TGF-β type 2 receptor (TGFBR2) siRNAs #1 (sense: 5′-CAACGGUGCAGUCAAGUUUUU-3′; antisense: 5′-AAACUUGACUGCACCGUUGUU-3′; Genolution), and TGFBR2 siRNAs #2 (sense: 5′-CCAAUAUCCUCGUGAAGAAUU-3′; antisense: 5′-UUCUUCACGAGGAUAUUGGUU-3′; Genolution). Mixtures with equal amounts of TGFBR1 siRNAs #1 and #2, and TGFBR2 siRNAs #1 and #2, were used to knockdown TGFBR1 and TGFBR2, respectively.

### Western blot analysis

The cultured cells were washed twice with ice-cold PBS. They were incubated with 2× SDS buffer (100 mM Tris-HCl [pH 6.8], 2% [w/v] SDS, 0.01% bromophenol blue, 20% glycerol, and 10% β-mercaptoethanol) for 5 min at 25 °C. The cells were then collected by scraping with cell scrapers and proteins were denatured at 95 °C for 5 min. Western blot analysis was performed using the following primary antibodies: anti‐phospho‐Smad 2/3 (1:1,000; Cell Signaling Technology, Danvers, MA, USA), anti‐Smad2 (1:1,000; Cell Signaling Technology), anti-N-cadherin (1:2,000, Invitrogen), anti‐phospho-ERKs (1:5,000; Cell Signaling Technology), anti‐ERKs (1:2,000; Cell Signaling Technology), anti‐phospho‐Akt (1:4,000; Cell Signaling Technology), anti‐Akt (1:1,000; Cell Signaling Technology), anti‐phospho‐p38 (1:1,000; Cell Signaling Technology), anti‐p38 (1:1,000; Cell Signaling Technology), anti-Smad4 (1:1,000; Cell Signaling Technology), anti-OB-cadherin (1:1,000; Cell Signaling Technology), and α‐tubulin (1:20,000; Sigma). Densitometry of the bands obtained was performed with the ImageJ software (NIH, Bethesda, MD, USA).

### Quantitative real-time polymerase chain reaction (qRT-PCR)

Total RNA was extracted using the TRIzol reagent (Invitrogen, Carlsbad, CA, USA), and cDNA was synthesized using the SuperScript III Reverse Transcriptase (Invitrogen), according to the manufacturer's protocol. Quantitative real-time reverse transcriptase-polymerase chain reaction (qRT-PCR) was performed using the Power SYBR Green PCR Master Mix (Invitrogen). Human ribosomal protein S9 gene (RPS9) was used as an endogenous control. The primers used to detect the expression levels of RPS9, N-cadherin, OB-cadherin, Smad4, TGF-β1, TGFBR1, and TGFBR2 were as follows: RPS9 (sense): 5′-CTGACGCTTGATGAGAAGGAC-3′, RPS9 (antisense): 5′-CAGCTTCATCTTGCCCTCAT-3′; N-cadherin (sense): 5′-CATCCCTCCAATCAACTTGC-3′, N-cadherin (antisense): 5′-ATGTGCCCTCAAATGAAACC-3′; OB-cadherin (sense): 5′-CCAACAGCCCGATAAGGTAT-3′, OB-cadherin (antisense): 5′-TGGATTTCTGCTGCAAAGAC-3′; Smad4 (sense): 5′-ATCTATGCCCGTCTCTGGAGGT-3′, Smad4 (antisense): 5′-CAGGAATGTTGGGAAAGTTGGC-3′; TGF-β1 (sense): 5′-CAACACATCAGAGCTCCGAGAA-3′, TGF-β1 (antisense): 5′-AAGGCGAAAGCCCTCAATTT-3′; TGFBR1 (sense): 5′-ATTACCAACTGCCTTATTATGA-3′, TGFBR1 (antisense): 5′-CATTACTCTCAAGGCTTCAC-3′; TGFBR2 (sense): 5′-GTCTACTCCATGGCTCTGGT-3′, TGFBR2 (antisense): 5′-ATCTGGATGCCCTGGTGGTT-3′.

### Immunocytochemistry

BM-MSCs were plated at a density of 1 × 10^4^ cells/cm^2^ on type 1 collagen (Nitta Gelatin Inc., Osaka, Japan)-coated cover slips in 24-well plates and cultured in the culture medium for 24 h. Thereafter, the medium was replaced with serum-free DMEM supplemented with 1% L-glutamine and 1% P/S and the cells were cultured for another 18 h. The cells were then treated with TGF-β for 24 h, and subsequently fixed with 4% PFA (Sigma) in PBS for 10 min on ice. After fixation, immunocytochemistry was performed according to standard protocols. The fixed cells were washed with PBS three times and permeabilized with 0.2% Triton-X 100 (Sigma) in PBS for 5 min at 25 °C. The cells were again washed three times with 0.1% Triton-X 100 in PBS and blocked with 5% skimmed milk (Becton, Dickinson and Company, Franklin Lakes, NJ, USA) prepared in 0.1% Triton-X 100, for 30 min at 25 °C. After blocking, the cells were incubated for 1 h 30 min with the following primary antibodies: anti-N-cadherin (1:30, Invitrogen) and anti-β-catenin (1:100, Bethyl Laboratories, Montgomery, TX, USA). Next, the cells were washed three times with 1% skimmed milk in 0.1% Triton-X 100 and incubated with secondary antibodies for 30 min at 25 °C. If necessary, actin was stained with Alexa Fluor 546-tagged phalloidin (1:1,000, Invitrogen). After incubation with secondary antibody, the cells were washed three times with PBS, and nuclei were stained using 4,6‐diamidino‐2‐phenylindole (DAPI, Invitrogen) for 10 min. The cells were then mounted using Fluoromount-G solution (SouthernBiotech, Birmingham, AL, USA). Images were taken using a Zeiss LSM 700 confocal microscope (Carl Zeiss, Oberkochen, Germany). The fluorescence intensity of N-cadherin-positive structures at the borders between BM-MSCs was measured using the ImageJ software (NIH).

### Transwell migration assay

BM-MSCs were incubated with DMEM supplemented with 2% FBS, 1% L-glutamine, and 1% P/S for 18 h. Millicell culture plate inserts (8 μm pore size; EMD Millipore) were coated with Type I collagen (5 μg/mL; Nitta Gelatin NA Inc.). The serum-starved BM-MSCs were seeded at a density of 2 × 10^4^ cells/cm^2^ in the upper chamber of each insert using DMEM supplemented with 2% FBS, 1% L-glutamine, and 1% P/S and incubated for 6 h. Thereafter, the media in both the lower and upper chamber of each insert were replaced with serum-free DMEM supplemented with 1% L-glutamine and 1% P/S. TGF-β (10 ng/mL) or solvent was added to the lower chamber of each well. If necessary, cells were treated with SB505124 (500 nM) or EGTA (4 mM) for 30 min prior to treatment with TGF-β. The cells were then incubated for 12 h, fixed with 4% PFA in PBS for 2 h at 25 °C, washed with PBS, and then stained using DAPI (Invitrogen) for 10 min. The membranes were mounted using Fluoromount-G solution (SouthernBiotech). Cells on either the lower or upper surface of five randomly selected areas of each Millicell membrane were imaged using a Zeiss LSM 700 confocal microscope (Carl Zeiss). The number of cells in each image was counted using Adobe Photoshop CS6 (Adobe Systems Incorporated, San Jose, CA, USA), and the number of migrated cells was determined as a percentage of total cells on both sides of the insert. To evaluate the migration of BM-MSCs toward breast cancer cells, CM from MDA-MB-231 and MCF7, or the control CM were added to the lower chamber of each insert.

### Three-dimensional (3D) cell migration assay

BM-MSCs were suspended at a density of 5 × 10^5^ cells/mL in 100 μL of a mixture (5:1:1:3) of Type 1A collagen solution (final concentration, 1.5 mg/mL [Nitta Gelatin Inc.]), reconstitution buffer (2.2 g NaHCO_3_ and 200 mM HEPES in 0.05 N NaOH), 10× Minimum Essential Medium (Invitrogen), and sterile ultrapure distilled water. Three microliters of the cell-collagen mixture was solidified in 12-well plates and incubated with serum-free DMEM supplemented with 1% L-glutamine and 1% P/S for 6 h. The media were then changed with the CM from MDA-MB-231 or the control. The migration of BM-MSCs was observed after 24 h. The migration of BM-MSCs was determined by dividing the area occupied by cells migrated from each gel drop by the area occupied by non-migrated cells in the outer edge corresponding to 10% of each drop prior to adding the CM. Images were captured using a Nikon ECLIPSE TS 100 inverted microscope (Nikon Instruments Inc., Melville, NY, USA), and the area was measured using the ImageJ software (NIH). A cell-collagen mixture containing BM-MSCs transfected with N-cadherin siRNA and BM-MSCs transfected with the control siRNA was used to evaluate the effects of N-cadherin on cell-cell adhesion between migrating BM-MSCs. BM-MSCs transfected with the control siRNA were stained with calcein AM before mixing. The cell-collagen mixture was solidified on a μ-dish 35 mm, low Grid-500 (ibidi, Munich, Germany). Migrating cells on the surface of 13 randomly selected areas were imaged using a Zeiss LSM 700 confocal microscope (Carl Zeiss) to analyze the number of migrating control BM-MSCs and migrating N-cadherin knockdown BM-MSCs.

### Statistical analysis

Quantitative data are expressed as the mean or mean ± SD. Statistical significance was analyzed using the Student's *t*-test. The GraphPad version 6.07 (GraphPad Software, Inc. San Diego, CA, USA) was used for statistical analyses. A value of *P* < 0.05 was considered significant.

## Results

### TGF-β-induced migration of BM-MSCs is associated with the increase in N-cadherin

**Figure 1:**
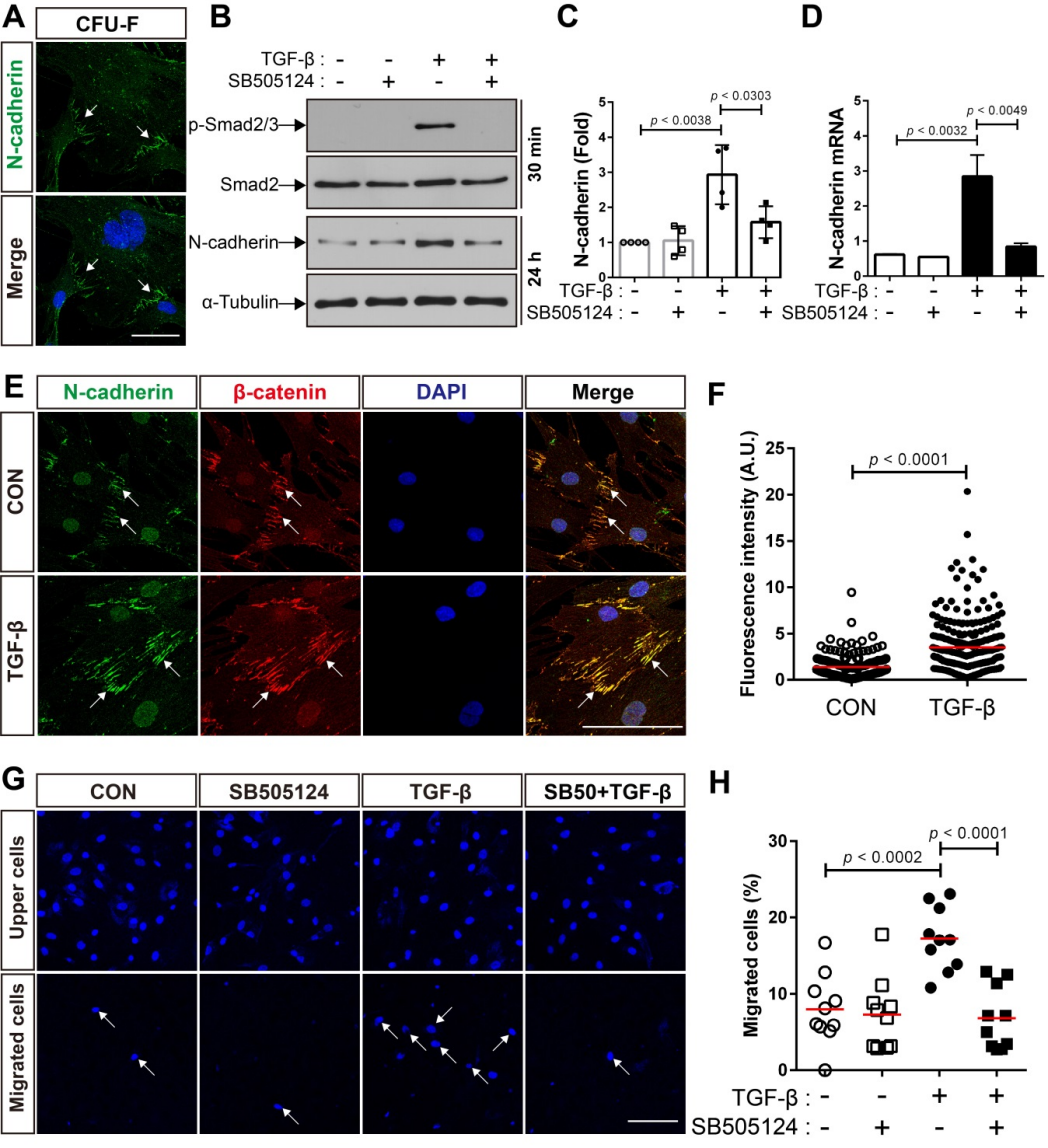
**TGF-β increases N-cadherin expression and migration of bone marrow-derived mesenchymal stem cells (BM-MSCs). (A)** Localization of N-cadherin on the borders of colony-forming cells. White arrows indicate positive signals for N-cadherin on the intercellular borders. **(B)** Western blot analysis of phosphorylated Smad2/3 (p-Smad2/3), Smad2, N-cadherin, and ɑ-tubulin in BM-MSCs treated with SB505124 (500 nM), an inhibitor of TGF-β type 1 receptor, for 30 min prior to treatment with TGF‐β (1 ng/mL) for the indicated time. **(C)** Densitometric analysis of western blot results for the expression of N-cadherin in BM-MSCs treated with SB505124 (500 nM) for 30 min prior to treatment with TGF‐β (1 ng/mL) for 24 h (results are presented as the mean ± SD; four independent experiments). **(D)** Expression level of N-cadherin mRNA in BM-MSCs treated with SB505124 (500 nM) for 30 min prior to treatment with TGF‐β (1 ng/mL) for 24 h (results are presented as the mean ± SD). **(E-F)** Immunocytochemistry of BM-MSCs treated with TGF‐β (1 ng/mL) or vehicle (CON) for 24 h. Nuclei were stained with DAPI in blue and white arrows indicate co-localization of N-cadherin and β‐catenin at intercellular borders. Quantification of fluorescence intensity of N-cadherin signals in **(F)**. The red lines indicate the mean values (*n* = 3 samples for each group). **(G-H)** Quantification of BM-MSCs migrated in response to TGF‐β (10 ng/mL) after pretreatment with SB505124 (500 nM) for 30 min. White arrows indicate DAPI-stained nuclei of migrated BM-MSCs on the lower membrane surface. Quantification of migrated BM-MSCs in **(H)**. The red lines indicate the mean values (*n* = 2 samples for each group). Scale bars indicate 100 μm.

We first investigated whether MSCs isolated from adult mouse bone marrow express N-cadherin. A suspension of bone marrow cells was prepared by treating crushed long bones of adult mice with collagenase, and the cells were allowed to form colonies. All the colony-forming MSCs expressed N-cadherin on the border between the cells inside the colonies **(Figure 1A)**. It is likely that N-cadherin may contribute to the formation of cell-cell adhesion-like structures between BM-MSCs. In a previous study, we demonstrated that N-cadherin mediates the migration of human BM-MSCs in response to TGF-β 48. We examined whether the expression of N-cadherin is regulated by TGF-β. TGF-β increased the expression of N-cadherin at both the protein and mRNA levels in BM-MSCs as well as the phosphorylation of Smad2/3; however, SB505124, an inhibitor of TGF-β type 1 receptor, inhibited the TGF-β-induced increase in N-cadherin expression and phosphorylation of Smad2/3 **(Figure 1B-D)**. Interestingly, TGF-β induced only a slight increase in the expression of OB-cadherin, another major cadherin in BM-MSCs, compared with the expression of N-cadherin **(Figure S1A-B)**. Therefore, N-cadherin may be a major cadherin that is induced in BM-MSCs by TGF-β. N-cadherin, induced by TGF-β, was concentrated and localized at the border between BM-MSCs, and β-catenin co-localized with N-cadherin at the borders **(Figure 1E-F)**. These results suggest that TGF-β-induced N-cadherin may be concentrated at the borders between BM-MSCs and may contribute to the formation of cell-cell adhesion-like structures of BM-MSCs, which are similar to adherens junctions of epithelial and endothelial cells. Importantly, TGF-β induced the migration of BM-MSCs via the TGF-β type 1 receptor-mediated signaling. TGF-β increased the migration of BM-MSCs, but SB505124 inhibited their migration toward TGF-β **(Figure 1G-H)**.

### N-cadherin is required for the migration of BM-MSCs in response to TGF-β

**Figure 2:**
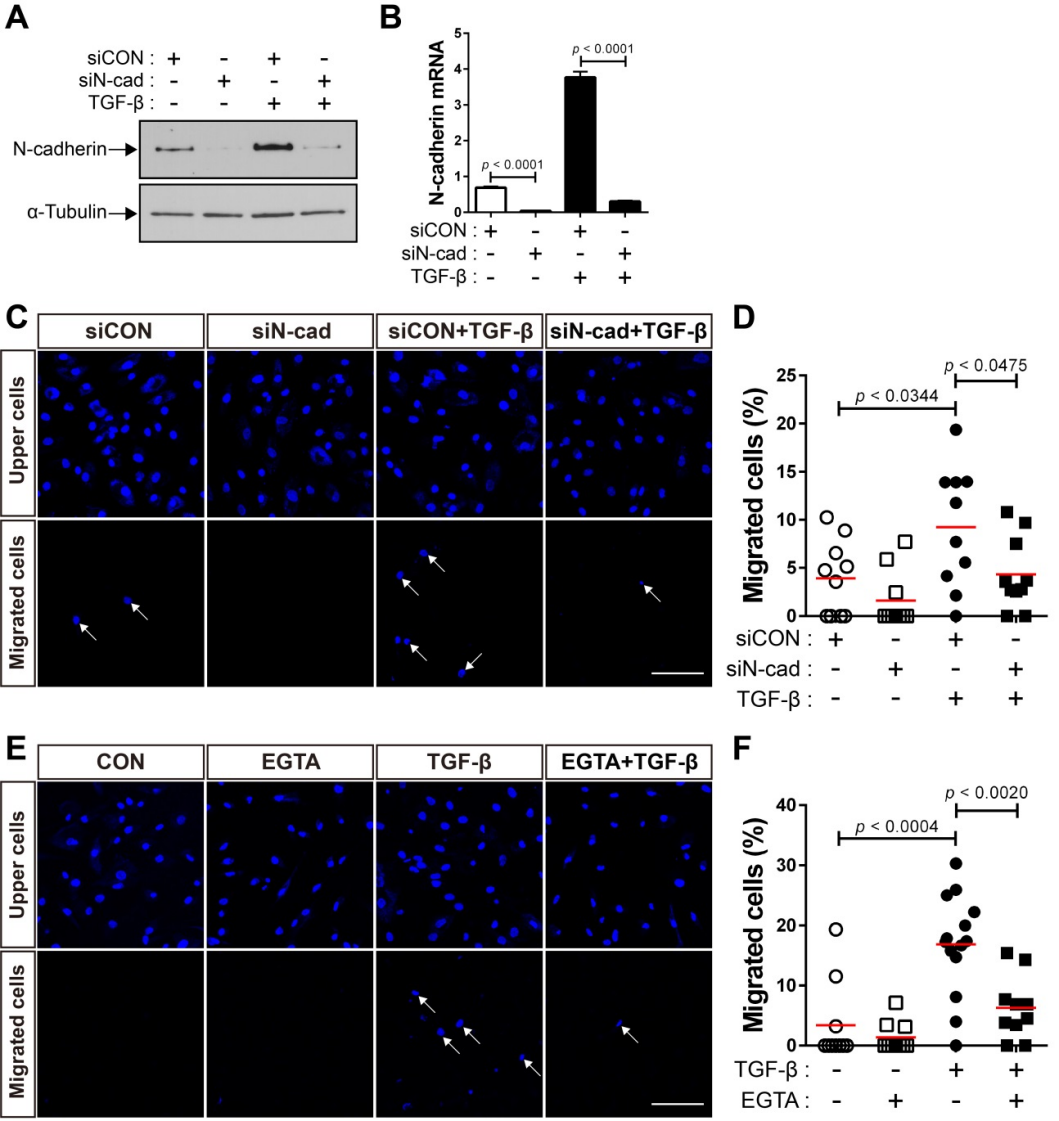
**N-cadherin is necessary for TGF-β-mediated migration of bone marrow-derived mesenchymal stem cells (BM-MSCs). (A-B)** Western blot analysis of N-cadherin and ɑ-tubulin and qRT-PCR analysis of N-cadherin. BM-MSCs transfected with control siRNA (siCON) or N‐cadherin siRNA (siN‐cad) were treated with TGF‐β (1 ng/mL) for 24 h. Results are presented as mean ± SD. **(C-D)** Migration of BM-MSCs transfected with each siRNA in response to TGF‐β (10 ng/mL). **(E-F)** Migration of BM-MSCs pretreated with EGTA for 30 min in response to TGF‐β (10 ng/mL). White arrows indicate DAPI-stained nuclei of migrated BM-MSCs on the lower membrane surface. The red lines indicate the mean values (*n* = 2 samples for each group). Scale bars indicate 100 μm.

Next, we performed siRNA-mediated knockdown of N‐cadherin to investigate whether the upregulation of N-cadherin by TGF-β results in the migration of BM-MSCs to TGF-β. Compared with the control siRNA, transfection with N‐cadherin siRNA decreased the expression levels of N‐cadherin at the protein and mRNA levels **(Figure 2A-B)**. The knockdown of N‐cadherin downregulated the migration of BM-MSCs toward TGF-β **(Figure 2C-D)**. EGTA, a high-affinity chelator of calcium ions, which are essential for homophilic *trans*-interactions of N-cadherin, also downregulated the migration of BM-MSCs toward TGF-β **(Figure 2E-F)**. These results suggest that the intercellular interaction of BM-MSCs, mediated by N-cadherin, upregulated by TGF-β, is required for their migration toward TGF-β.

### TGF-β enhances the expression of N-cadherin in BM-MSCs via Smad4

**Figure 3:**
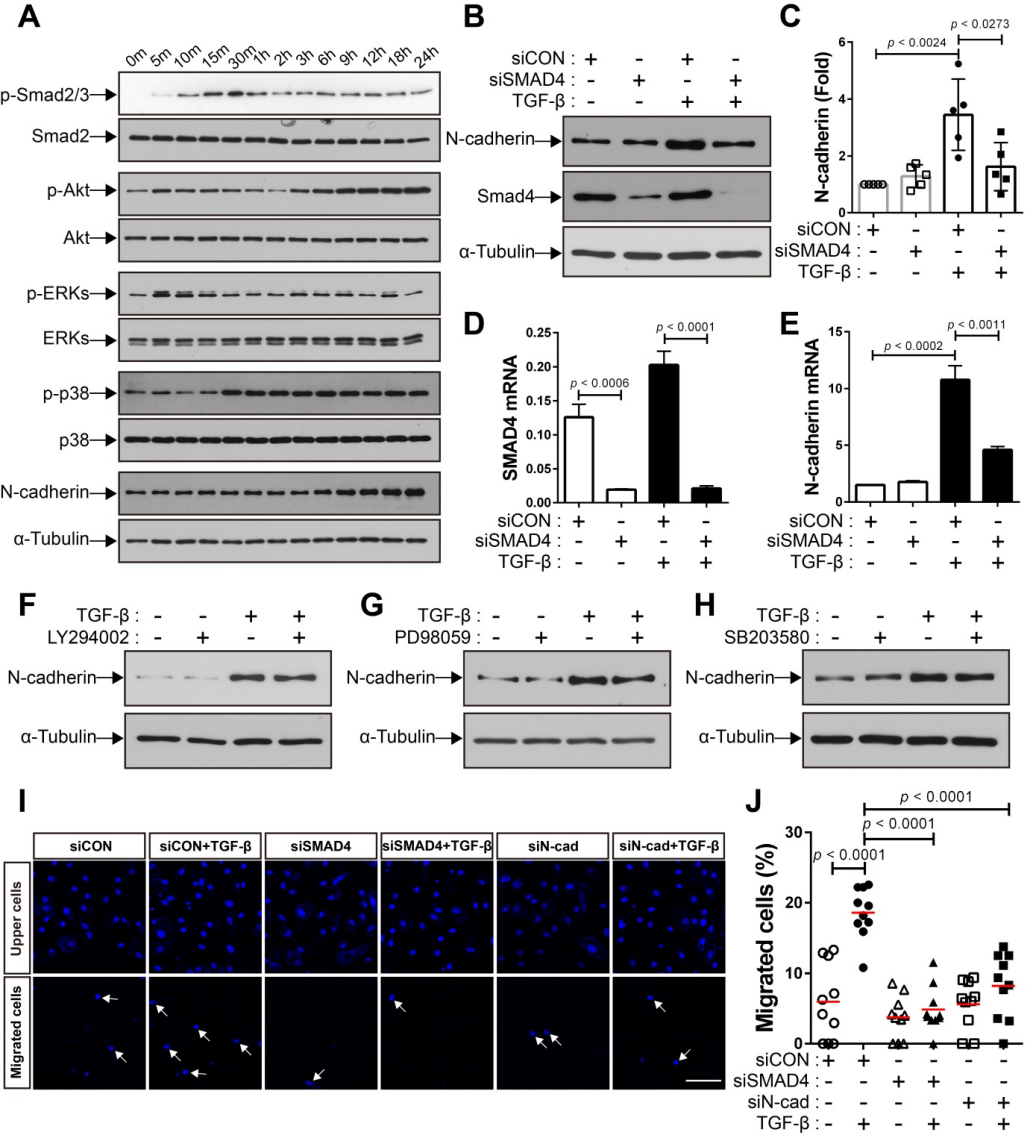
**TGF-β increases N-cadherin expression in bone marrow-derived mesenchymal stem cells (BM-MSCs) in a Smad4-dependent manner. (A)** Western blot analysis of phosphorylated Smad2/3 (p-Smad2/3), Smad2, phosphorylated Akt (p-Akt), Akt, phosphorylated ERKs (p-ERKs), ERKs, phosphorylated p38 (p-p38), p38, N-cadherin, and ɑ-tubulin. BM‐MSCs were treated with TGF‐β (1 ng/mL) for the indicated times. **(B-C)** Western blot analysis of N-cadherin, Smad4, and ɑ-tubulin. BM-MSCs transfected with control siRNA (siCON) or Smad4 siRNA (siSMAD4) were treated with TGF‐β (1 ng/mL) for 24 h. Densitometric analysis of western blot results in **(C)**. Results are presented as mean ± SD (five independent experiments). **(D-E)** qRT-PCR analysis of Smad4 and N-cadherin. BM-MSCs transfected with each siRNA were treated with TGF‐β (1 ng/mL) for 24 h. **(F-H)** Western blot analysis of N-cadherin and ɑ-tubulin. BM-MSCs were pretreated with LY294002 (2 μM), PD98059 (10 μM), or SB203580 (10 μM) for 30 min prior to treatment with TGF-β (1 ng/mL) for 24 h. **(I-J)** Migration of BM-MSCs transfected with each siRNA in response to TGF‐β (10 ng/mL). White arrows indicate DAPI-stained nuclei of migrated BM-MSCs on the lower membrane surface. Quantification of the results of migration assay in **(J)**. The red lines indicate the mean values (*n* = 2 samples for each group). Scale bar indicates 100 μm.

We investigated the mechanisms underlying the increase in the expression of N-cadherin in BM-MSCs in response to TGF-β. TGF-β activates non-canonical signaling pathways, including Akt, ERKs, and p38, as well as canonical signaling, as shown in our previous study 48. TGF‐β induced Smad 2/3 phosphorylation in BM-MSCs as early as 10 min post-treatment, and the highest phosphorylation level was observed at 30 min post-treatment, followed by a gradual decrease in the level of Smad 2/3 phosphorylation at later time points **(Figure 3A)**. The phosphorylation of Akt, ERK, and p38 was upregulated in BM-MSCs in response to TGF‐β **(Figure 3A)**. TGF‐β gradually increased the expression of N-cadherin **(Figure 3A)**. The TGF‐β-mediated canonical signaling is dependent on Smad4 21. siRNA-mediated knockdown of Smad4 significantly inhibited the increase in the expression of N-cadherin induced by TGF‐β **(Figure 3B-E)**. However, this TGF‐β-induced increase was slightly affected by inhibitors of TGF‐β non-canonical signaling. Pretreatment with LY294002 (a specific inhibitor of phosphoinositol 3‐kinase, upstream of Akt), PD98059 (a specific inhibitor of mitogen‐activated protein kinase kinase, upstream of ERKs), or SB203580 (a specific inhibitor of p38) did not inhibit the increase in the expression of N-cadherin in BM-MSCs **(Figure 3F-H)**. Importantly, TGF‐β-mediated migration of BM-MSCs was reduced by the knockdown of Smad4, to a similar extent as in the case of N-cadherin knockdown **(Figure 3I-J)**. Therefore, these results suggest that N-cadherin increases in response to TGF‐β via Smad4 and mediates the migration of BM-MSCs toward TGF‐β.

### N-cadherin mediates the migration of BM-MSCs in response to breast tumor cells

**Figure 4:**
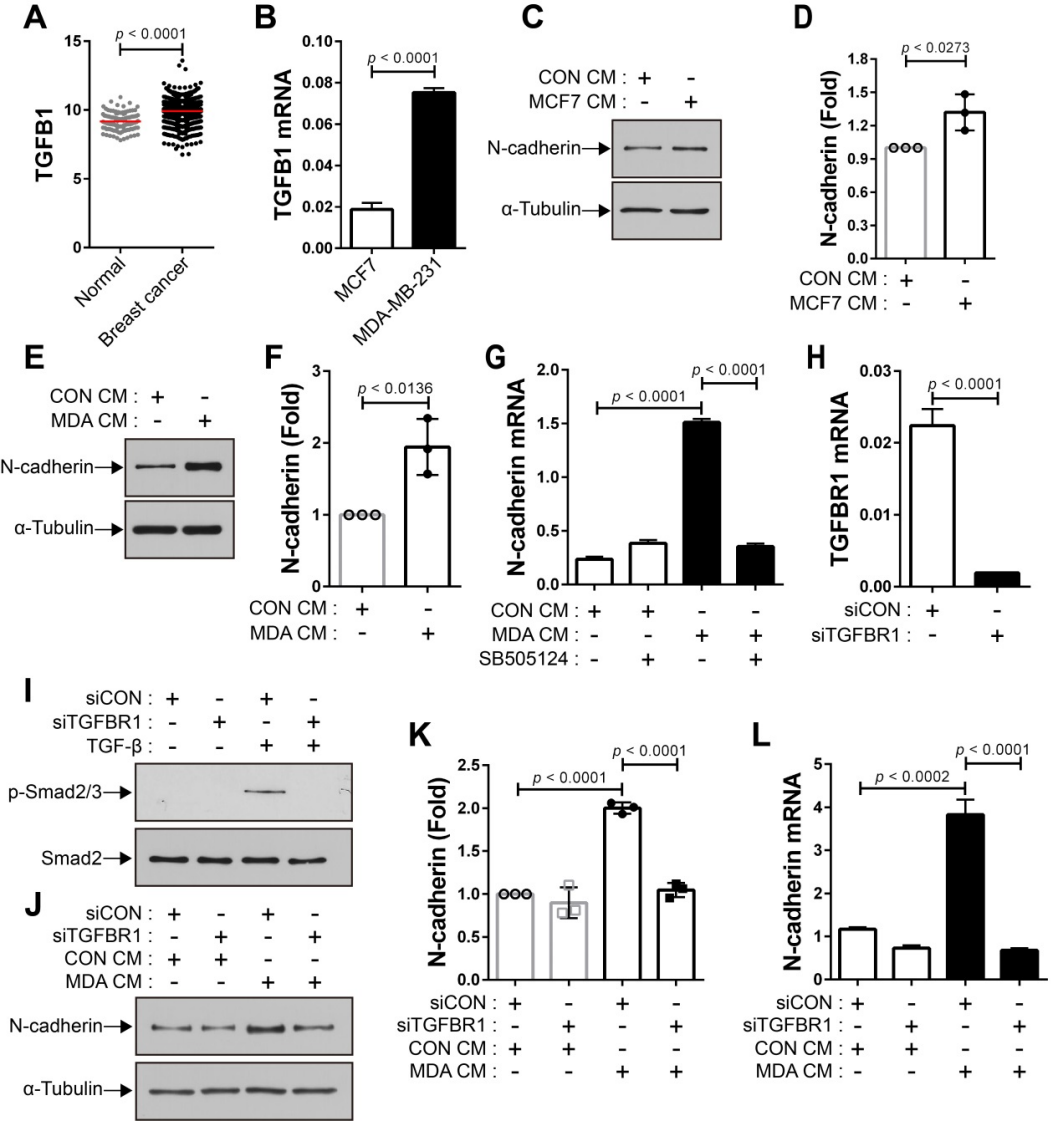
**Breast tumor cell-conditioned medium increases the expression of N-cadherin in bone marrow-derived mesenchymal stem cells (BM-MSCs) in a TGF-β-dependent manner. (A)** The expression of TGF-β1 ligand in breast cancer compared with that in the normal tissue. The RNA-seq database of human breast cancers from The Cancer Genome Atlas (TCGA) was analyzed. The red lines indicate the mean values. **(B)** qRT-PCR analysis of TGFB1, a gene of TGF-β1 ligand in MCF7 and MDA-MB-231 cells. **(C-D)** Western blot analysis of N-cadherin and ɑ-tubulin. BM-MSCs were treated with the control conditioned medium (CON CM) or MCF7 conditioned medium (MCF7 CM) for 24 h. Densitometric analysis of western blot results in (three independent experiments) in **(D)**. **(E-F)** Western blot analysis of N-cadherin and ɑ-tubulin. BM-MSCs were treated with CON CM or the MDA-MB-231 conditioned medium (MDA CM) for 24 h. Densitometric analysis of western blot results (three independent experiments) in **(F)**. **(G)** qRT-PCR analysis of N-cadherin. BM-MSCs were treated with SB505124 (500 nM) for 30 min prior to treatment with CON CM or MDA CM for 24 h. **(H)** qRT-PCR analysis of TGFBR1, a gene of TGF-β type 1 receptor in BM-MSCs to verify the knockdown of TGFBR1. **(I)** Western blot analysis of phosphorylated Smad2/3 (p-Smad2/3) and Smad2. BM-MSCs transfected with control siRNA (siCON) or TGF-β type 1 receptor siRNA (siTGFBR1) were treated with TGF‐β (1 ng/mL) for 30 min. **(J-K)** Western blot analysis of N-cadherin and ɑ-tubulin. BM-MSCs transfected with each siRNA were treated with CON CM or MDA CM for 24 h. Densitometric analysis of western blot results (three independent experiments) in **(K)**. **(L)** qRT-PCR analysis of N-cadherin. BM-MSCs transfected with each siRNA were treated with CON CM or MDA CM for 24 h. Results are presented as the mean ± SD.

**Figure 5:**
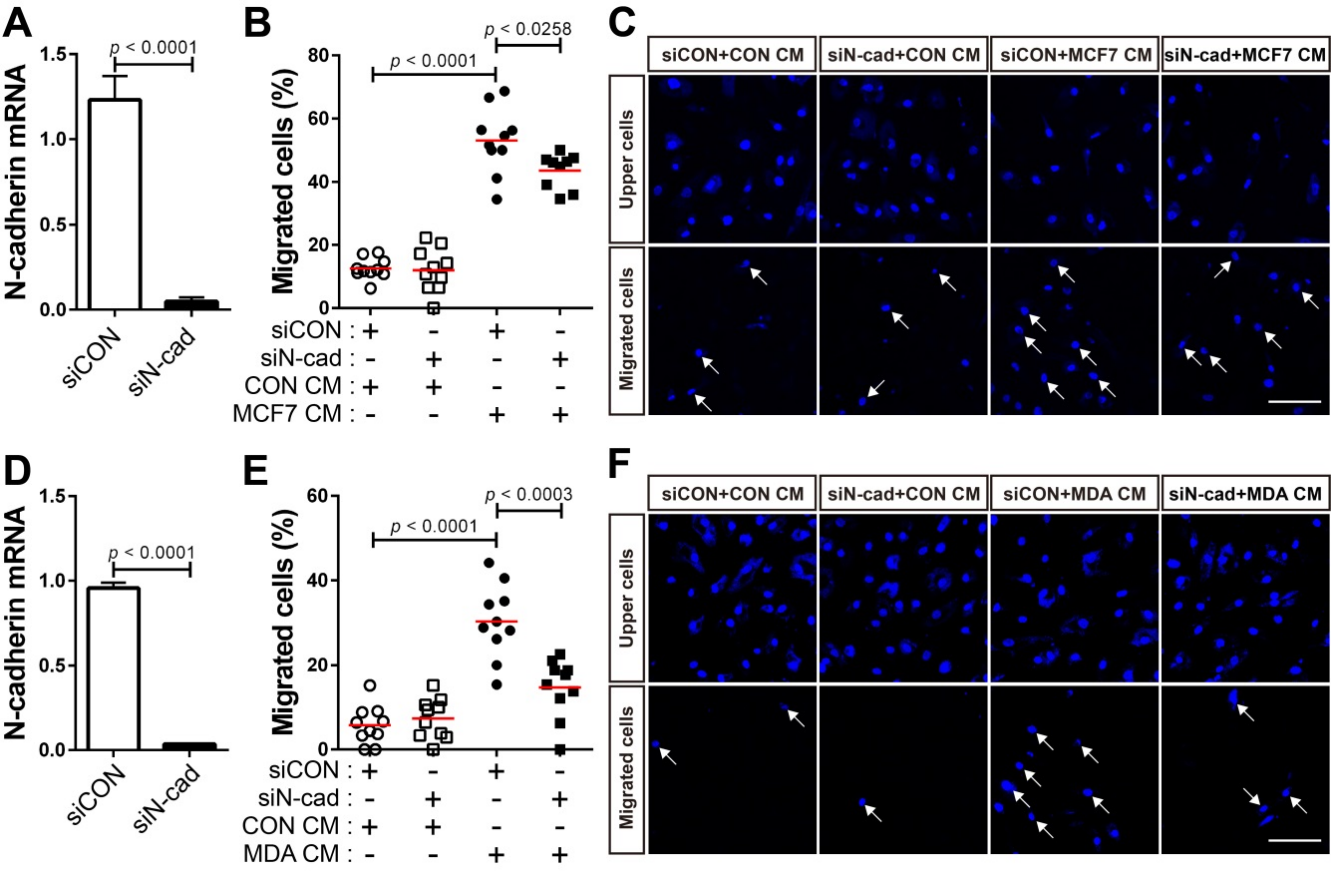
**N-cadherin mediates the migration of bone marrow-derived mesenchymal stem cells (BM-MSCs) toward MCF-7 or MDA-MB-231 conditioned medium. (A)** Verification of N-cadherin knockdown in BM-MSCs transfected with siRNA that were used in the migration assay with the MCF7 conditioned medium (MCF7 CM). **(B-C)** Migration of BM-MSCs transfected with each siRNA in response to MCF7 CM. BM-MSCs transfected with control siRNA (siCON) or N-cadherin siRNA (siN-cad) were treated with the control conditioned medium (CON CM) or MCF7 CM for 12 h. **(D)** Verification of N-cadherin knockdown in BM-MSCs transfected with siRNA that were used in the migration assay with the MDA-MB-231 conditioned medium (MDA CM). **(E-F)** Migration of BM-MSCs transfected with each siRNA in response to MDA CM. BM-MSCs transfected with each siRNA were treated with CON CM or MDA CM for 12 h. White arrows indicate DAPI-stained nuclei of migrated cells on the lower membrane surface. The red lines indicate the mean values (*n* = 2 samples for each group). Scale bars indicate 100 μm.

The expression of TGF-β was seen to be higher in human breast cancer tissue than in the normal tissue **(Figure 4A)**. There are conflicting reports about the tumor suppressor and tumor-promoting functions of TGF-β in breast cancer 24, 31, 54-58 and the expression of TGF-β is independent of the subtypes of human breast tumor cell lines (Cancer Cell Line Encyclopedia; CCLE). We examined whether breast cancer cells expressing TGF-β can modulate the motility of BM-MSCs via the regulation of N-cadherin expression. MCF-7 and MDA-MB-231 cells expressed TGF-β **(Figure 4B** and CCLE**)** 59, although the expression levels were different in the two cell lines. The expression of N-cadherin increased in BM-MSCs in response to MCF7 CM and MDA CM, compared with that in response to CON CM **(Figure 4C-F)**. However, the expression level of OB-cadherin did not change in response to MDA CM **(Figure S1C)**. MDA CM induced a higher level of N-cadherin in BM-MSCs than MCF7 CM **(Figure 4C-F**; 2-fold increase in response to MDA CM vs. 1.3-fold increase in response to MCF7 CM**)**. The difference in the expression of N-cadherin in BM-MSCs in response to MCF-7 CM and MDA CM was associated with the different expression levels of TGF-β in the two cell lines **(Figure 4B**; 4-times higher in MDA-MB-231 than in MCF-7**)**. Indeed, inhibition of TGF-β signaling affected the induction of N-cadherin in BM-MSCs. SB505124 impaired the upregulation of N-cadherin expression by MDA CM **(Figure 4G)**. SB431542, another inhibitor of TGF-β type 1 receptor, also decreased N-cadherin upregulation induced by MDA CM **(Figure S2A-B).** siRNA-mediated knockdown of TGF-β type 1 inhibited the phosphorylation of Smad2/3 in BM-MSCs in response to TGF-β **(Figure 4H-I)**. The knockdown of TGF-β type 1 receptor or TGF-β type 2 receptor inhibited the MDA CM-induced increase in N-cadherin expression in BM-MSCs **(Figure 4J-L; Figure S2C-E)**. BM-MSCs were able to migrate toward MCF7 CM and MDA CM. More importantly, the knockdown of N-cadherin downregulated the migration of BM-MSCs toward MCF7 CM **(Figure 5A-C)** or MDA CM **(Figure 5D-F)** as it inhibited their migration in response to TGF-β. We also found that BM-MSCs significantly enhanced the migration of MDA-MB-231 cells (**Figure S3**). These results suggest that BM-MSCs that migrate toward breast tumor N-cadherin-dependently enhance invasion and migration of breast tumor cells.

### N-cadherin mediates cell-cell adhesion required for the collective migration of BM-MSCs in response to breast tumor cells

**Figure 6:**
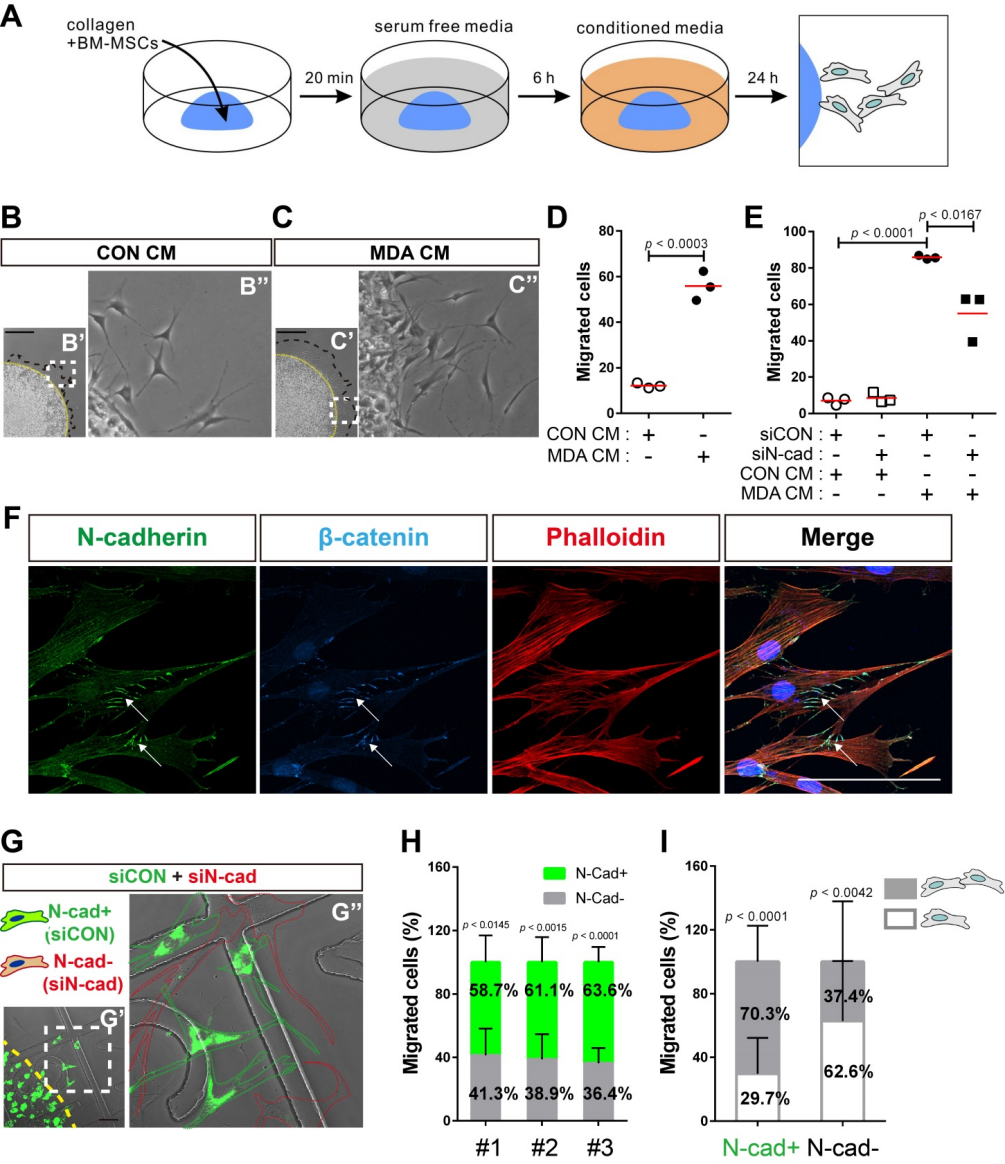
**N-cadherin-mediated cell-cell adhesion is required for the migration of bone marrow-derived mesenchymal stem cells (BM-MSCs) toward MDA-MB-231 conditioned medium. (A)** Schematic representation of three-dimensional (3D) migration assay. **(B-D)** Three-dimensional migration of BM-MSCs toward the control conditioned medium (CON CM) or MDA-MB-231 conditioned medium (MDA CM). White dashed boxes in B' and C' are magnified in B'' and C''. Yellow broken lines indicate the margin of collagen gel and black broken lines represent the leading edge of migrating cells. Scale bars indicate 600 μm. Quantification of the results in **(D)**. The red lines indicate the mean values (*n* = 3 samples for each group). **(E)** Three-dimensional migration of BM-MSCs transfected with control siRNA (siCON) or N-cadherin siRNA (siN-cad). The cells were treated with CON CM or MDA CM for 24 h. The red lines indicate the mean values (*n* = 3 samples for each group). **(F)** Immunocytochemistry of the expression of N-cadherin and β-catenin in BM-MSCs undergoing 3D migration toward MDA CM. Actin was stained with phalloidin in red and nuclei were stained with DAPI in blue. White arrows indicate co-localization of N‐cadherin and β‐catenin at cell-cell adhesion borders of migrating BM-MSCs. Scale bar indicates 100 μm. **(G-I)** Three-dimensional migration using a mixture of BM-MSCs transfected with siCON (stained with calcein AM in green) or siN-cad. White dashed boxes in G' are magnified in G'' and yellow broken line indicates the margin of collagen gel. Green dotted lines appear around calcein AM stained BM-MSCs transfected with siCON and red dotted lines appear around BM-MSCs transfected with siN-cad. Scale bar indicates 100 μm. **(H)** Percentages of BM-MSCs transfected with siCON or BM-MSCs transfected with siN-cad among the total number of migrating BM-MSCs from each mixture of cell-collagen gel **(***n* = 3**)**. **(I)** The percentage of migrating cells maintaining cell-cell adhesion or migrating cells without cell-cell adhesion among the total number of migrating BM-MSCs transfected with siCON (N-cad+ cells) or the percentage of migrating cells maintaining cell-cell adhesion or migrating cells without cell-cell adhesion among the total number of migrating BM-MSCs transfected with siN-cads (N-cad- cells) (gray bars: the percentages of migrating cells with cell-cell adhesion; white bars: the percentages of migrating cell without cell-cell adhesion). Results are presented as the mean ± SD (*n* = 3 mixtures of cell-collagen gel).

Next, we examined the role of N-cadherin in the migration of BM-MSCs toward breast cancer cells. Cell migration was analyzed using a 3D cell migration assay 60, 61 to examine whether BM-MSCs collectively migrate in response to breast cancer cells and maintain cell-cell adhesion during migration **(Figure 6A)**. Indeed, BM-MSCs migrated collectively and maintained cell-cell adhesion. The collective migration of BM-MSCs increased in response to MDA CM treatment compared with that of the control, while they maintained cell-cell adhesion **(Figure 6B-D)**. However, the knockdown of N-cadherin downregulated the migration of BM-MSCs **(Figure 6E)**, suggesting that N-cadherin may contribute to cell-cell adhesion, which is required for the migration of BM-MSCs. The results of immunocytochemistry showed that N-cadherin and β-catenin co-localized at the cell-cell adhesion sites, the borders between the migrating BM-MSCs, in response to MDA CM **(Figure 6F)**. N-cadherin on the borders was linked to actin filaments in the cytoplasm **(Figure 6F)**. A 3D cell migration assay was performed using a mixture of control BM-MSCs expressing N-cadherin and BM-MSCs in which N-cadherin was knocked down. A lesser number of cells in which N-cadherin was knocked down migrated in response to MDA CM than the control cells with intact expression of N-cadherin **(Figure 6G-H)**. Although some populations of BM-MSCs in which N-cadherin was knocked down still migrated in response to MDA CM, more than 60% of such BM-MSCs showed cell-cell adhesion-free migration. In contrast, less than 30% of the migrating cells expressing N-cadherin showed cell-cell adhesion-free migration **(Figure 6I)**. Overall, these results suggest that N-cadherin is required for cell-cell adhesion for mediating the collective migration of BM-MSCs toward breast tumor cells.

## Discussion

Cadherins are transmembrane adhesive molecules of adhesion junctions that control cell-cell adhesion, cell sorting, tissue dynamics, cellular metabolism, and cellular migration, and interact with various signal transducer proteins. N-cadherin is a critical regulator of collective migration of cells. However, it remains unclear whether N-cadherin contributes to cell-cell adhesion-dependent collective migration of BM-MSCs in response to various signals, including tumor-derived factors, such as TGF-β. In a previous study, we demonstrated that N-cadherin mediates the migration of BM-MSCs toward TGF-β. In the present study, we demonstrate that the expression of N-cadherin in BM-MSCs increases in a manner dependent on TGF-β canonical signaling (Smad4-dependent manner), and that N-cadherin-dependent cell-cell adhesion is required for the migration of BM-MSCs *in vitro* in response to TGF-β and breast tumor cells expressing TGF-β.

Adherens junctions, the dynamic cooperative cell-cell adhesion complexes, are required for the collective migration of mesenchymal cells, including BM-MSCs 62-64. The cell-cell adhesion mediated by adherens junctions consisting of cadherins mechanically links one cell to another and regulates their motility and protrusive behavior so as to mediate coordinated migration of mesenchymal cell cohorts. The coordinated collective migration of mesenchymal cells, such as the migration of the neural crest and head mesendoderm, has been found in embryonic development 62, 64. We demonstrate that cell-cell adhesion consisting of N-cadherin mediates the collective migration of BM-MSCs to MDA CM, an experimental condition simulating the tumor microenvironment derived by TGF-β-secreting breast tumor cells, but the mechanism(s) by which N-cadherin-dependent cell-cell adhesion mediates the collective migration of BM-MSCs remains unknown. N-cadherin-dependent cell-cell adhesion is required for the collective chemotactic migration of neural crest cells toward stromal cell-derived factor 1 (SDF-1). For migration, N-cadherin at the cell-cell contacts inhibits Rac1 activity and protrusion and in turn enhances the Rac1 activity and protrusion at the free edge 64. N-cadherin-dependent intercellular interaction also mediates the collective migration of glial cells 65 and of hepatocyte growth factor-treated MDCK cells 66 via the regulation of focal adhesion and dynamic regulation of the interaction between N-cadherin and ɑ-catenin, respectively. Further experiments are required to identify the mechanisms by which N-cadherin-dependent cell-cell adhesion mediates the collective migration of BM-MSCs toward breast tumors.

BM-MSCs express both OB-cadherin and N-cadherin. TGF-β and CM from breast tumor cells expressing TGF-β increased the expression of N-cadherin, but not that of OB-cadherin. As reported in our previous study, N-cadherin mediates the response of murine bone marrow-derived mesenchymal stem cell-like ST2 cells to TGF-β, although the expression level of N-cadherin in ST2 cells is not affected by TGF-β treatment 48. Therefore, further experiments are required to determine whether the migration of BM-MSCs is also regulated in an OB-cadherin-dependent manner. It has been found that the expression level of N-cadherin is tightly regulated for the directional invasive migration of tumor glial cells 65. Low levels of N-cadherin in glial cells cause less directional migration of the cells and accompany the formation of smaller focal adhesion points. Therefore, it is important to unravel the mechanisms underlying the increase in the expression of N-cadherin in BM-MSCs induced by TGF-β. TGF-β ligands intrinsically transduce canonical signaling in BM-MSCs in a manner dependent on Smad4 and non-canonical signaling independently of Smad4. Both TGF-β canonical and non-canonical signaling pathways were found to be required for the migration of BM-MSCs **(Figure 4** and unpublished**)** 48. There was a significant increase in the expression of N-cadherin mRNA 3 h after TGF-β treatment of BM-MSCs (unpublished). The knockdown of Smad4 inhibited TGF-β-induced increase in the expression of N-cadherin in BM-MSCs. In contrast, inhibitors of TGF-β non-canonical signaling pathways, such as those involving Akt, ERKs, and p38, did not appear to affect the expression of N-cadherin in BM-MSCs in response to TGF-β. However, further investigations might be necessary to examine whether the inhibitors downregulate the respective non-canonical signaling pathways. Importantly, TGF-β enhances the expression of N-cadherin in lung cancer cells by binding the Smad 2/3 complex to the N-cadherin promoter 67. The knockdown of Smad4 downregulated the expression of N-cadherin at mRNA and protein levels in TGF-β-treated BM-MSCs. Therefore, Smad protein complexes containing Smad4 may activate the promoter of N-cadherin in BM-MSCs to increase the expression of N-cadherin in response to TGF-β. It is also necessary to investigate whether canonical and/or non-canonical TGF-β signaling mediates the increase in N-cadherin in BM-MSCs in response to breast tumor-derived TGF-β. Further experiments are required to investigate these possibilities.

In this study, we introduce a promising novel strategy for re-engineering the breast tumor microenvironment. Homophilic *trans*-interactions of N-cadherin can be targeted to inhibit the recruitment of BM-MSCs into the breast tumor microenvironment that promotes metastasis and invasion of breast tumor cells and angiogenesis.

## Supplementary Material

Supplementary figures and tables.

## Abbreviations

BM-MSCs: bone marrow-derived mesenchymal stem cells
TGF-β: transforming growth factor β
N-cadherin: neural cadherin
ERKs: extracellular signal-regulated kinases
MCF7 CM: conditioned medium from MCF7
MDA CM: conditioned medium from MDA-MB-231
