# Supplementary material for: N-cadherin mediates the migration of bone marrow-derived mesenchymal stem cells toward breast tumor cells
Source: Theranostics. 2021 May 3;11(14):6786–99. doi: 10.7150/thno.59703 (PMC8171089; doi:10.7150/thno.59703)
Supplement: Supplementary file 1 — Supplementary figures and tables. [file thnov11p6786s1.pdf]

N-cadherin mediates the migration of bone marrow-derived mesenchymal stem cells toward breast tumor cells

Sanghyuk Choi[1], Jinyeong Yu[1], Wootak Kim[2], Ki-Sook Park[2,3*]

1. Graduate School of Biotechnology, Kyung Hee University, Yongin 17104, Republic of Korea.

2. Department of Biomedical Science and Technology, Graduate School, Kyung Hee University, Seoul 02447, Republic of Korea.

3. East-West Medical Research Institute, Kyung Hee University, Seoul 02447, Republic of Korea.

*Corresponding author: Ki-Sook Park, E-mail: kisookpark@khu.ac.kr

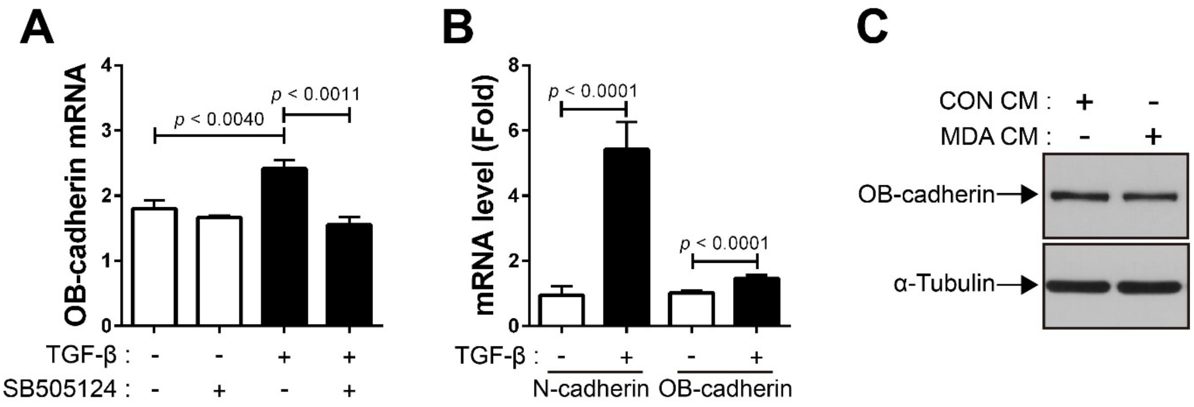

Figure S1. OB-cadherin is not significantly increased in bone marrow-derived mesenchymal stem cells (BM-MSCs) in response to TGF-β and MDA-MB-231 conditioned medium, compared with N-cadherin. (A) qRT-PCR analysis of OB-cadherin. BM-MSCs were treated with SB505124 (500 nM) for 30 min prior to treatment with TGF-β for 24 h. **(B)** qRT-PCR analysis of N-cadherin and OB-cadherin using BM-MSCs treated with TGF-β for 24 h. **(C)** Western blot analysis of OB-cadherin and α-tubulin. BM-MSCs were treated with the control conditioned medium (CON CM) or MDA-MB-231 conditioned medium (MDA CM) for 24 h.

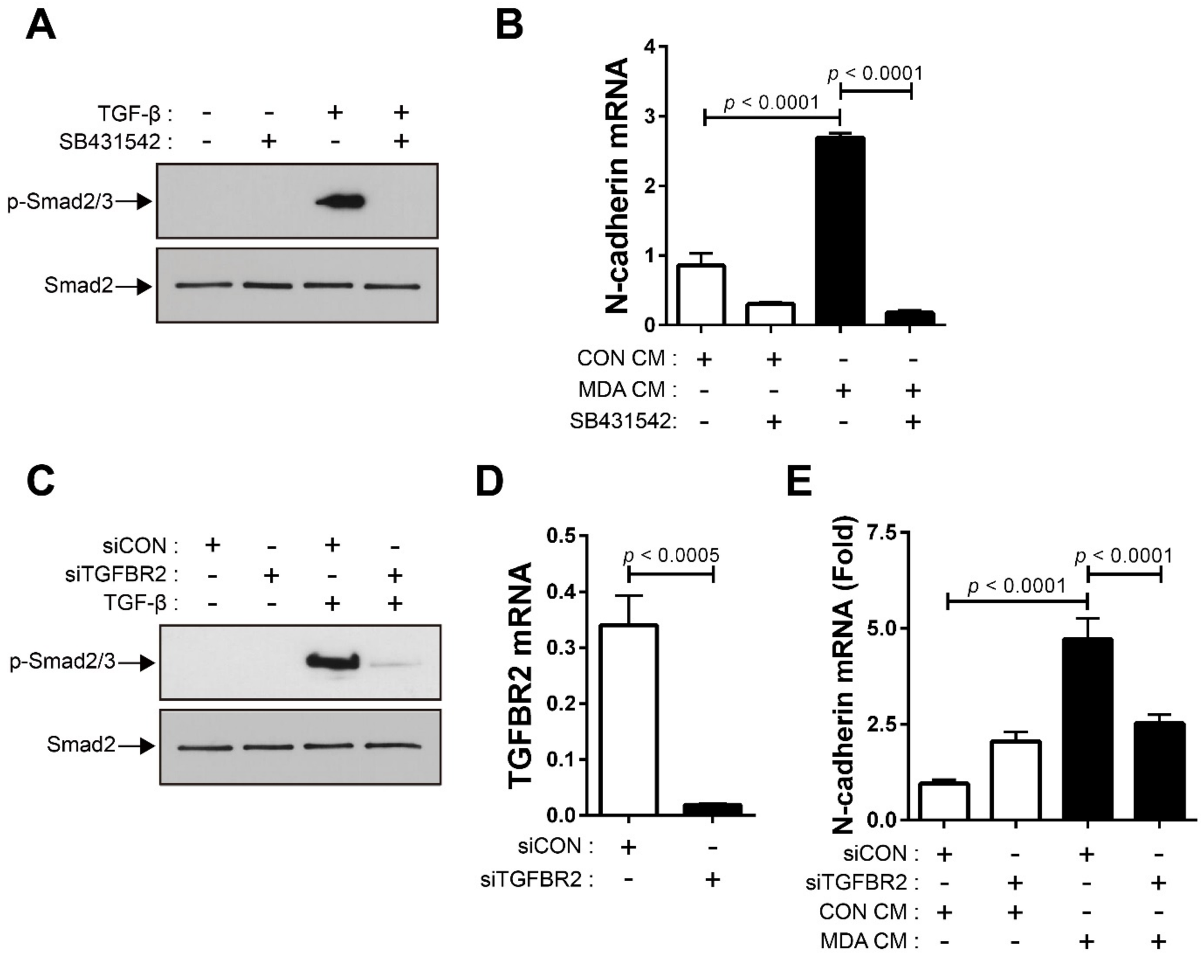

Figure S2. SB431542 and TGFBR2 knockdown inhibit the increase in N-cadherin mRNA expression induced by MDA-MB-231 conditioned medium. (A) Verification of the inhibition of TGF-β type 1 receptor-dependent signaling by SB431542; western blot analysis of phosphorylated Smad2/3 (p-Smad2/3) and Smad2 in BM-MSCs pretreated with SB431542 (10 μM) for 30 min prior to treatment with TGF-β (1 ng/mL) for 30 min. **(B)** Expression level of N-cadherin mRNA in BM-MSCs treated with SB431542 (10 μM) for 30 min prior to treatment with the control conditioned medium (CON CM) or MDA-MB-231 conditioned medium (MDA CM) for 24 h. **(C)** Verification of the inhibition of TGF-β signaling in response to knockdown of TGF-β type 2 receptor; western blot analysis of phosphorylated Smad2/3 (p-Smad2/3) and Smad2. BM-MSCs transfected with control siRNA (siCON) or TGF-β type 2 receptor siRNA (siTGFBR2) were treated with TGF-β (1 ng/mL) for 30 min. **(D)** qRT-PCR analysis of TGFBR2, gene encoding TGF-β type 2 receptor in BM-MSCs, to verify the knockdown of TGFBR2. (**L)** qRT-PCR analysis of N-cadherin. BM-MSCs transfected with each siRNA were treated with CON CM or MDA CM for 24 h. Results are presented as mean ± SD.

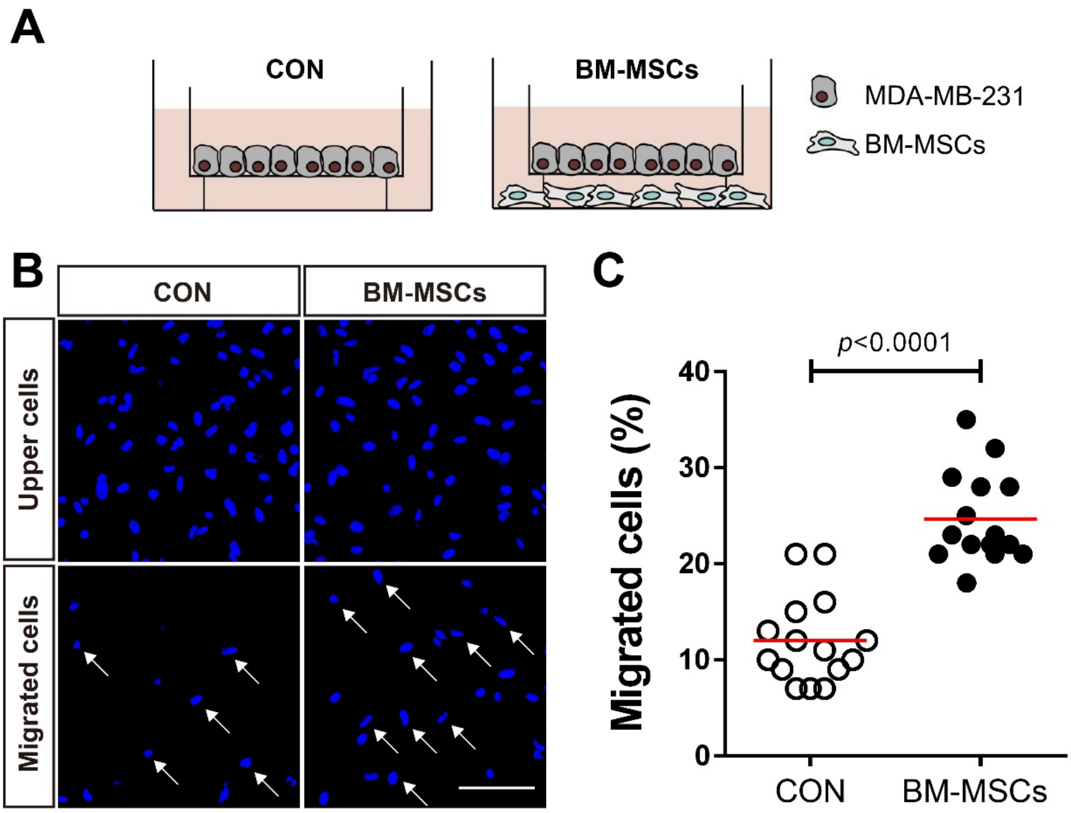

Figure S3. BM-MSCs promote the migration of MDA-MB-231 cells. (A) Schematic representation of MDA-MB-231 cells co-cultured with BM-MSCs using Millicell. Serum-starved MDA-MB-231 cells (5×10^4 cells) were seeded on the upper membrane of each Millicell (pore size, 8 μm) with the medium (1% FBS). After incubation for 6 h, MDA-MB-231-seeded Millicell inserts were transferred to 24-well plates, where BM-MSCs were cultured with the medium (1% FBS) or not. The migration of MDA-MB-231 was assessed 12 h later. **(B-C)** Migration of MDA-MB-231 cells in response to BM-MSCs. White arrows indicate DAPI-stained nuclei of migrated cells on the lower membrane surface. **(C)** Quantification of the results of migration assay. The red lines indicate the mean values (n = 2 samples for each group). Scale bars indicate 100 μm.
